# Accelerometer-derived movement features as predictive biomarkers for muscle atrophy in neurocritical care: a prospective cohort study

**DOI:** 10.1186/s13054-024-05067-y

**Published:** 2024-08-31

**Authors:** Moritz L. Schmidbauer, Timon Putz, Leon Gehri, Luka Ratkovic, Andreas Maskos, Julia Zibold, Johanna Bauchmüller, Sophie Imhof, Thomas Weig, Max Wuehr, Konstantinos Dimitriadis

**Affiliations:** 1 Department of Neurology, LMU University Hospital, LMU Munich, Munich, Germany; 2 Department of Anaesthesiology, LMU University Hospital, LMU Munich, Munich, Germany; 3 German Center for Vertigo and Balance Disorders (DSGZ), LMU University Hospital, LMU Munich, Munich, Germany

**Keywords:** ICU, ICUAW, Sarcopenia, Muscle atrophy, Accelerometer, Machine learning

## Abstract

**Background:**

Physical inactivity and subsequent muscle atrophy are highly prevalent in neurocritical care and are recognized as key mechanisms underlying intensive care unit acquired weakness (ICUAW). The lack of quantifiable biomarkers for inactivity complicates the assessment of its relative importance compared to other conditions under the syndromic diagnosis of ICUAW. We hypothesize that active movement, as opposed to passive movement without active patient participation, can serve as a valid proxy for activity and may help predict muscle atrophy. To test this hypothesis, we utilized non-invasive, body-fixed accelerometers to compute measures of active movement and subsequently developed a machine learning model to predict muscle atrophy.

**Methods:**

This study was conducted as a single-center, prospective, observational cohort study as part of the MINCE registry (metabolism and nutrition in neurointensive care, DRKS-ID: DRKS00031472). Atrophy of rectus femoris muscle (RFM) relative to baseline (day 0) was evaluated at days 3, 7 and 10 after intensive care unit (ICU) admission and served as the dependent variable in a generalized linear mixed model with Least Absolute Shrinkage and Selection Operator regularization and nested-cross validation.

**Results:**

Out of 407 patients screened, 53 patients (age: 59.2 years (SD 15.9), 31 (58.5%) male) with a total of 91 available accelerometer datasets were enrolled. RFM thickness changed − 19.5% (SD 12.0) by day 10. Out of 12 demographic, clinical, nutritional and accelerometer-derived variables, baseline RFM muscle mass (beta − 5.1, 95% CI − 7.9 to − 3.8) and proportion of active movement (% activity) (beta 1.6, 95% CI 0.1 to 4.9) were selected as significant predictors of muscle atrophy. Including movement features into the prediction model substantially improved performance on an unseen test data set (including movement features: R^2^ = 79%; excluding movement features: R^2^ = 55%).

**Conclusion:**

Active movement, as measured with thigh-fixed accelerometers, is a key risk factor for muscle atrophy in neurocritical care patients. Quantifiable biomarkers reflecting the level of activity can support more precise phenotyping of ICUAW and may direct tailored interventions to support activity in the ICU. Studies addressing the external validity of these findings beyond the neurointensive care unit are warranted.

**Trial registration:**

DRKS00031472, retrospectively registered on 13.03.2023.

**Supplementary Information:**

The online version contains supplementary material available at 10.1186/s13054-024-05067-y.

## Background

Intensive care unit acquired weakness (ICUAW) describes a neuromuscular dysfunction secondary to critical illness and its treatment with consecutive generalized weakness. Data on prevalence for ICUAW show considerable variation due to diverse patient demographics and heterogenous methodology. However, with a systematic review pinpointing the median prevalence at 43% [1], its ubiquity in critical care is evident. Moreover, the impact resulting from ICUAW is profound and long-lasting, with patient outcomes significantly compromised for up to five years after discharge [2–6]. Therefore, ICUAW is acknowledged as a key component of post intensive care syndrome (PICS), highlighting its importance in the continuum of long-term recovery following critical care [7, 8].

ICUAW needs to be recognized as a clinical syndrome, rather than a specific disease entity. As such, it exhibits great heterogeneity and partially overlapping pathologies, which has diluted research findings and made the identification of treatable targets challenging in the past [9–12]. Relevant and common entities include critical illness myopathy (CIM), critical illness polyneuropathy (CIP) as well as critical illness polyneuromyopathy (CIPNM) as an overlap syndrome [9, 11, 12]. Electrophysiological methods including nerve conduction studies (NCS), electromyography and direct muscle stimulation have been successfully used to establish biomarkers for CIM, CIP and CIPMN [9, 13, 14]. Muscle atrophy due to mechanical unloading is also being recognized as a critical component of ICUAW. However, measurable biomarkers to assess the extent of inactivity of muscles are lacking.

In this regard, it is important to note that activity arises from active movement, as opposed to passive movement during mobilization without active patient participation. Hence, we postulate that establishing a proxy for activity can be achieved by applying non-invasive, body-fixed accelerometers to the lower extremities of critically ill patients while prospectively excluding episodes with passive mobilization such as intrahospital transports, physiotherapy and patient positioning. By introducing these biomarkers as continuous measures of active movement and incorporating these variables into a machine learning model, we aimed to predict rectus femoris muscle atrophy, as measured by ultrasound up to day 10 of intensive care unit (ICU) treatment. Based on the hypothesis that neurocritical care patients exhibit a higher prevalence of inactivity due to disorders of consciousness and motor deficits, we specifically included patients with acute brain injury in this trial.

## Methods

### Study design, setting and clinical management

This study was designed as a single-center, prospective, observational cohort study as part of the MINCE registry (metabolism and nutrition in neurointensive care, DRKS-ID: DRKS00031472, retrospectively registered on 13.03.2023) at a tertiary academic center (LMU University Hospital, Munich, Germany). Reporting follows the Strengthening the Reporting of Observational Studies in Epidemiology (STROBE) reporting guidelines. This study was approved by the local ethics committee (LMU Munich, project number 22-0173, 11.04.2022). Written consent was obtained from all participants or their next of kin. The study recruited from April 2022 to March 2024 and included patients within 48 h after ICU admission with age ≥ 18 years, neurologic disease as admitting diagnosis, and expected ICU length of stay ≥ 10 days. Patients with pre-existing neuromuscular disease, renal replacement therapy, pregnancy, pre-existing neoplastic disease, recent hospitalization (hospital stays longer than three days in the last three months, ICU treatment within the last three months), pre-existing confinement to bed, and pre-existing frailty (Clinical Frailty Scale > 3) were excluded.

Patients were mobilized at the treating physicians’ discretion. If indicated, patients received physiotherapy for 20–40 min/day on six days of the week and were repositioned and transferred from bed to chair regularly by the nursing staff. Nutritional therapy was conducted according to the European Society of Parenteral and Enteral Nutrition (ESPEN) guidelines, with caloric and protein targets of 25 kcal/kg/day and 1.3 g/kg/day, respectively [15]. As a reference, body weight as measured with bed scales was used for non-obese patients, and ideal body weight was used for patients with a body mass index (BMI) > 30 kg/m^2^ [15]. During the acute phase of illness (days 1–3), hypocaloric nutrition (70% of energy expenditure (EE)) was aimed for. From day 4 on, isocaloric (100% of EE) nutrition was implemented.

### Data collection

Clinical data prospectively collected on the ICU included age, sex, body mass index (BMI), admission diagnosis, cumulative protein and calorie deficit, duration of mechanical ventilation, ICU length of stay (LOS), daily Sepsis-related Organ Failure Assessment score (SOFA) and SOFA without Glasgow Coma Scale (GCS) score (mSOFA), Acute Physiology and Chronic Health Evaluation (APACHE II) score on ICU admission, Nutrition Risk in Critically ill score (NUTRIC) on admission, premorbid modified Rankin Scale (pmRS) and Glasgow Outcome Scale Extended (GOSE) at ICU discharge.

Ultrasound of the upper thigh (rectus femoris muscle, RFM) and temporalis muscle (TM) was performed bilaterally using a 20 MHz linear probe (MyLabOmega, Esaote, Genoa, Italy) upon admission, and on days 3, 7 and 10. As previously described [16, 17], the site of measurement for RFM was marked in the lower third of the connecting line between the anterior superior iliac spine and the upper edge of the patella with a permanent marker to ensure reproducibility between measurements (Supplementary Fig. 1). Measurements were conducted according to a local protocol that emphasized minimal compression during RFM sonography and called for individual adjustments of depth and gain to optimally visualize the surface of the femur and to delineate fascial borders, respectively. Measurement of TM followed the protocol as described by Maskos et al. [17] Three repeated measurements were performed by one of six raters (TP, LG, LR, AM, JB, SI), using the built-in software of the ultrasound machine to measure muscle thickness. The mean value of the repeated measurements was used for further analysis. Reliability of repeated ultrasound measurements is reported in Supplementary Table 1.

Tri-axial accelerometers (range ± 16 g; sampling rate 12.5 Hz; Axivity Ltd., Newcastle upon Tyne, UK) were attached within 48 h of admission to both upper thighs with transparent adhesive tape. Skin inspections and minor adjustments to the sensor placement were performed every third day to prevent any pressure damage. To exclude any passive movement not contributing to the patient’s activity, episodes with physiotherapy, intrahospital transports, or repositioning by nursing staff were prospectively documented and excluded from the recorded data. The sensor data was extracted and analyzed by an author (MW) not involved in the patients’ clinical management and blinded for the ultrasound measurements.

As a positive control, accelerometers were attached to healthy individuals (n = 3). Static placement of sensors served as a negative control (Supplementary Table 2).

### Accelerometer data processing

**Fig. 1:**
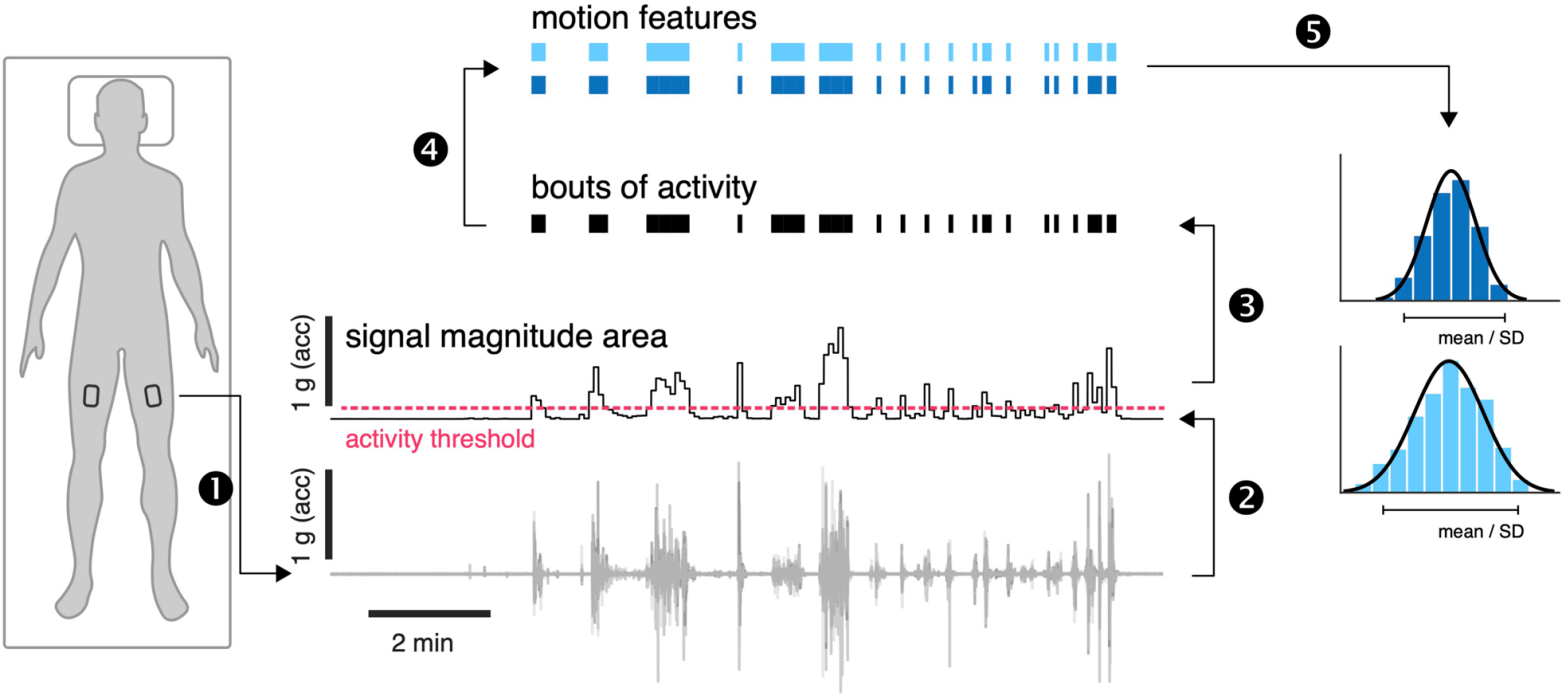
Accelerometer-derived features. Body motion was monitored using tri-axial accelerometers bilaterally attached to the upper thigh (1). Raw triaxial accelerometer recordings were first offset eliminated, and the time series were segmented into non-overlapping 5 s windows. The signal magnitude area was computed for every window (2). Bouts of dynamic activity were identified based on the threshold ≥ 0.135 g (3) and a set of motion features was computed for every bout of activity (4). Finally, the average and distribution of motion features across all bouts of activity were computed (5). acc = acceleration

OmGUI version 1.0.0.11 software was used to download the raw acceleration data from the devices. MATLAB (Mathworks Inc., Natick, USA, release R2022a, version 9.12.0) was used for data processing. Periods of active movement were identified following a previously established procedure that has been already used for activity recognition in ICU settings [18, 19]. Accordingly, recorded time series from each axial component (x, y, z) were first down-sampled to 10 Hz and subsequently high-pass filtered (4th order Butterworth filter, cutoff frequency at 0.2 Hz) to remove baseline offset and low-frequency effects reflecting static postural orientation. Filtered time series were segmented into non-overlapping 5 s windows for subsequent motion feature extraction. Signal magnitude area (SMA) was then computed for every window [19] to identify activity bouts (AB) using a defined threshold of SMA ≥ 0.135 g [18]. Across all identified AB, the mean intensity (AB-intensity) and duration (AB-duration) as well as the variability (standard deviation, SD) of these features were calculated. The distribution of motion features across ABs is log-normal, which required estimating mean and SD via a maximum likelihood technique [20]. The overall movement intensity, the proportion of active movement (%active) and ABs per hour were calculated based on the entire duration of the recording (Fig. 1).

### Predictive modeling and statistical analysis

As the dataset includes multiple observations (both legs) per patient and exhibits linearity as evaluated by exploratory data analysis, a generalized linear mixed model (GLMM) to account for intra-patient correlation by using individual patients as a random effect was chosen. Given the numerous independent variables of interest, including demographic, clinical, and activity-related features, a rigorous approach to model selection and validation to prevent overfitting was required. Therefore, we employed regularization with Least Absolute Shrinkage and Selection Operator (LASSO), which penalizes the GLMM model via L1-norm and in effect shrinks the weight of non-contributing features to zero.

**Fig. 2:**
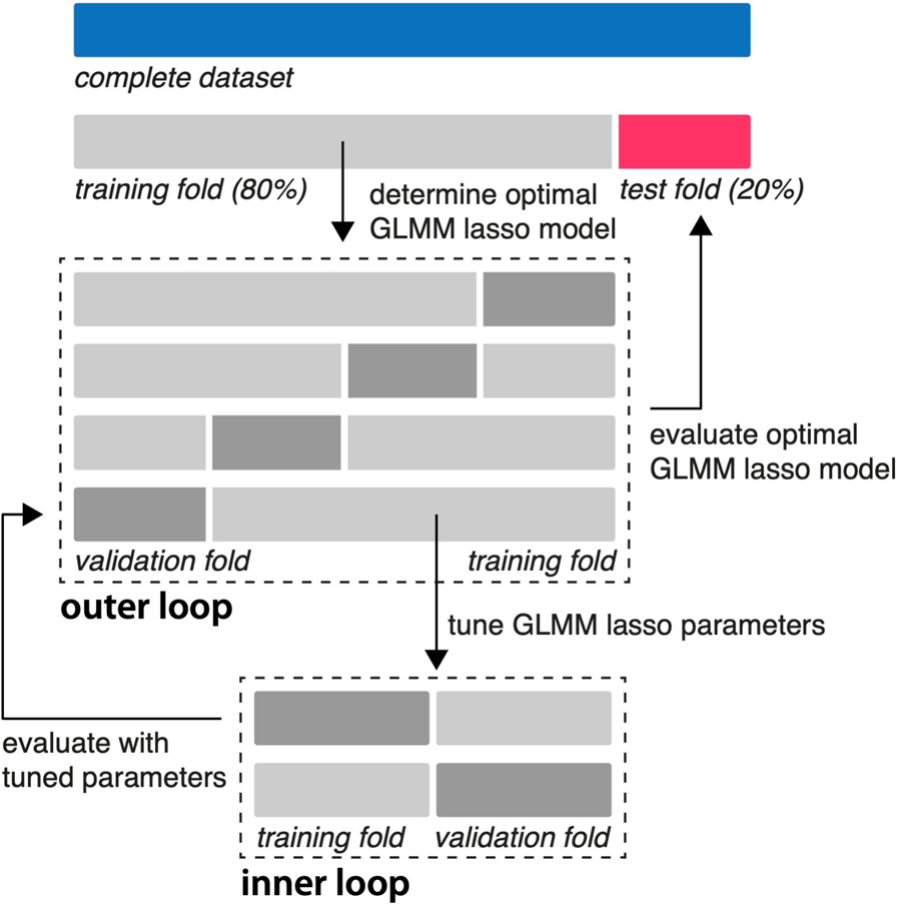
Nested-cross validation of a regularized GLMM model**.** After standardization and a stratified 80/20 split, the training data set was partitioned into 4 folds (outer loop). Within each outer fold, an inner loop of 2 folds was used for hyperparameter tuning. The hyperparameter (lambda) that minimized the mean squared error in the inner loop was selected. The model with this optimal lambda was then evaluated on the validation fold of the outer loop. This process was repeated for all 4 outer folds, resulting in an optimal lambda for each fold. The final model was chosen using the average of the optimal lambdas from all outer folds. Finally, the performance of this final model was assessed using the unseen test set. GLMM = generalized mixed effects model; lasso = least absolute shrinkage and selection operator

First, multicollinearity among predictors was mitigated by excluding variables with a variance inflation factor (VIF) exceeding 5 (removing AB per hour and SOFA) [21]. Next, standardization (z-score normalization) of the remaining prediction variables (age, sex, baseline RFM muscle mass, mSOFA, calorie deficit, protein deficit, overall intensity, %active, AB-intensity_log-mean_, AB-duration_log-mean_, AB-intensity_log-SD_, AB-duration_log-SD_) was performed to ensure equal weights and comparable units. To allow testing on unseen data, a stratified split was executed to divide the data into training (80%) and test (20%) sets. The training set was further used for optimizing the hyperparameter of GLMM-LASSO using a machine learning approach with nested cross-validation (Fig. 2) [22]. Model performance was evaluated on the test set using mean squared error, root mean squared error, mean absolute error, R-squared (squared correlation method, R2) and a plot depicting actual versus predicted values.

To illustrate the level of uncertainty of the model coefficients, bootstrapping with 10,000 resamples was performed to estimate the bias-corrected and accelerated (BCa) confidence intervals for the model coefficients.

Additionally, and to test the relevance of leg movement on TM as a muscle group unaffected by thigh movement, we used a linear regression model for the prediction of TM atrophy at day 10 including all demographic, clinical and nutritional variables as well as %active as independent variables. To compare %active between healthy individuals and the neurointensive care unit (NICU) cohort, a two-tailed t-test was performed. Further, we identified patients with unilateral upper motor neuron damage and corresponding motor deficits to investigate the contribution of upper motor neuron lesion on muscle atrophy. To compare the magnitude of muscle atrophy and to account for within-subject correlation, we used Generalized Estimating Equations (GEE) with post hoc pairwise comparisons and Bonferroni adjustment.

Summary statistics for continuous variables are presented as means with standard deviation (SD) for normally distributed data and as medians with interquartile ranges (IQR) for non-normally distributed data, with normality assessed using Quantile–Quantile plots and Shapiro–Wilk test. Categorical variables are summarized as frequencies and percentages.

All analyses were performed using R (2023.06.1 + 524) using ‘stats’, ‘psych’, ‘ggplot2’, ‘dplyr’, ‘lme4’, ‘nlme’, ‘geepack’, ‘multcomp’, ‘emmeans’, ‘MASS’, ‘svglite’ ‘glmmLasso’,‘caret’ and ‘boot’, packages. ChatGPT (version 4) was used for error handling, repetitive programming, and overall optimization of code in R.

## Results

### Patient characteristics

**Fig. 3:**
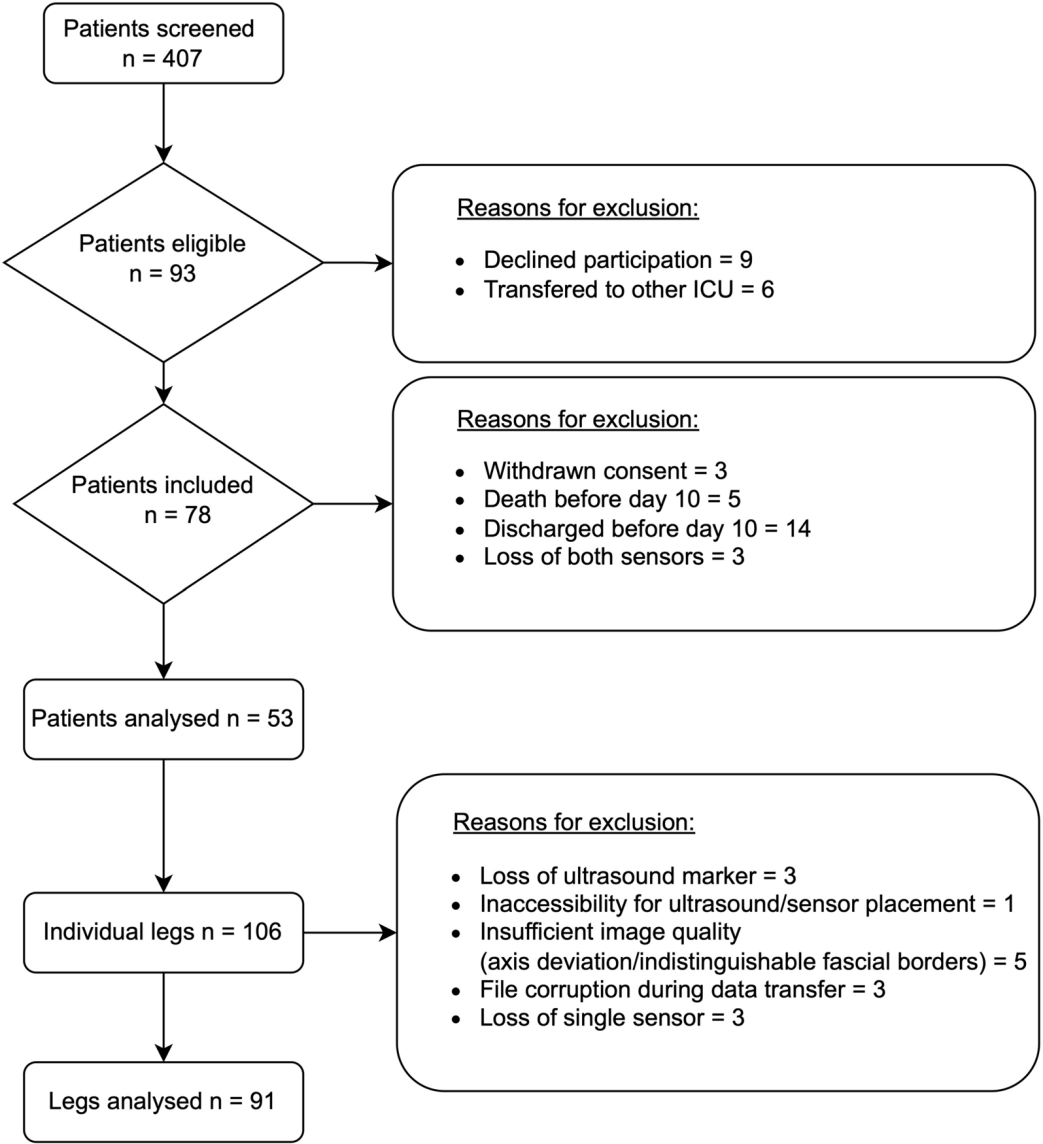
Screening and study inclusion. ICU = Intensive Care Unit;

Out of 407 patients screened, 53 with a total of 91 available accelerometer datasets were enrolled in this study (Fig. 3). Clinical baseline characteristics are presented in Table 1. Of all patients included in the analysis, mean age was 59.2 years (SD 15.9) and 31 (58.5%) were male. Cerebrovascular diseases were the most frequent ICU admission diagnoses (86.8%, 46/53). Mean ICU length of stay was 17.0 days (IQR 8.0), while the mean duration of mechanical ventilation was 15.6 days (SD 9.2). During the observation period, patients met 62.6% (SD 18.4) of the caloric goals and 57.9% (SD 21.6) of protein goals according to the ESPEN guidelines.

**Table 1:** Baseline characteristics

Parameter	n = 53
Age (years), mean (SD)	59.2 (15.9)
Male, n (%)	31 (58.5)
BMI (kg/m^2^), median (IQR)	27.2 (6.2)
Baseline RFM thickness (mm), mean (SD)	10.3 (2.6)
APACHE II score at ICU admission, median (IQR)	17.0 (4.5)
SOFA score (without GCS) at ICU admission, mean (SD)	4.5 (2.1)
NUTRIC score at ICU admission, mean (SD)	3.4 (2.0)
No disability at ICU admission (pmRS = 0), n (%)	40 (75.5)
pmRS = 1, n(%)	7 (13.2)
pmRS = 2, n(%)	6 (11.3)
ICU admission diagnosis, n (%)	
Intracerebral hemorrhage	21 (39.6)
Ischemic stroke	8 (15.0)
Subarachnoid hemorrhage, non-traumatic	17 (32.1)
Meningitis/Encephalitis/other neuro-infectious disease	3 (5.7)
Status epilepticus	2 (3.8)
Guillain-Barré syndrome	1 (1.9)
Cerebral venous sinus thrombosis	1 (1.9)
Medical nutritional management	
Calories administered/calories prescribed up to day 10, % [SD]	62.6 (18.4)
Protein administered/protein prescribed up to day 10, % [SD]	57.9 (21.6)
ICU admission to sensor placement (hours), median [IQR]	42.8 (10.3)
ICU LOS, days, median (IQR)	17.0 (8.0)
Duration of mechanical ventilation, days, mean (SD)	15.6 (9.1)
Hospital LOS, days, median (IQR)	21.0 (15.0)
ICU mortality, n (%)	5 (9.6)
GOSE at hospital discharge, median (IQR)	4 (3–5)

IQR = inter-quartile range; SD = standard deviation; ICU = intensive care unit; BMI = Body Mass Index; RFM = rectus femoris muscle; APACHE II = Acute Physiology and Chronic Health Evaluation; SOFA = Sequential organ failure assessment; NUTRIC = Nutrition Risk in Critically ill score; pmRS = premorbid modified Rankin Scale; LOS = length of stay; GOSE = Glasgow Outcome Scale—Extended

### Active movement and muscle atrophy during ICU treatment

**Fig. 4:**
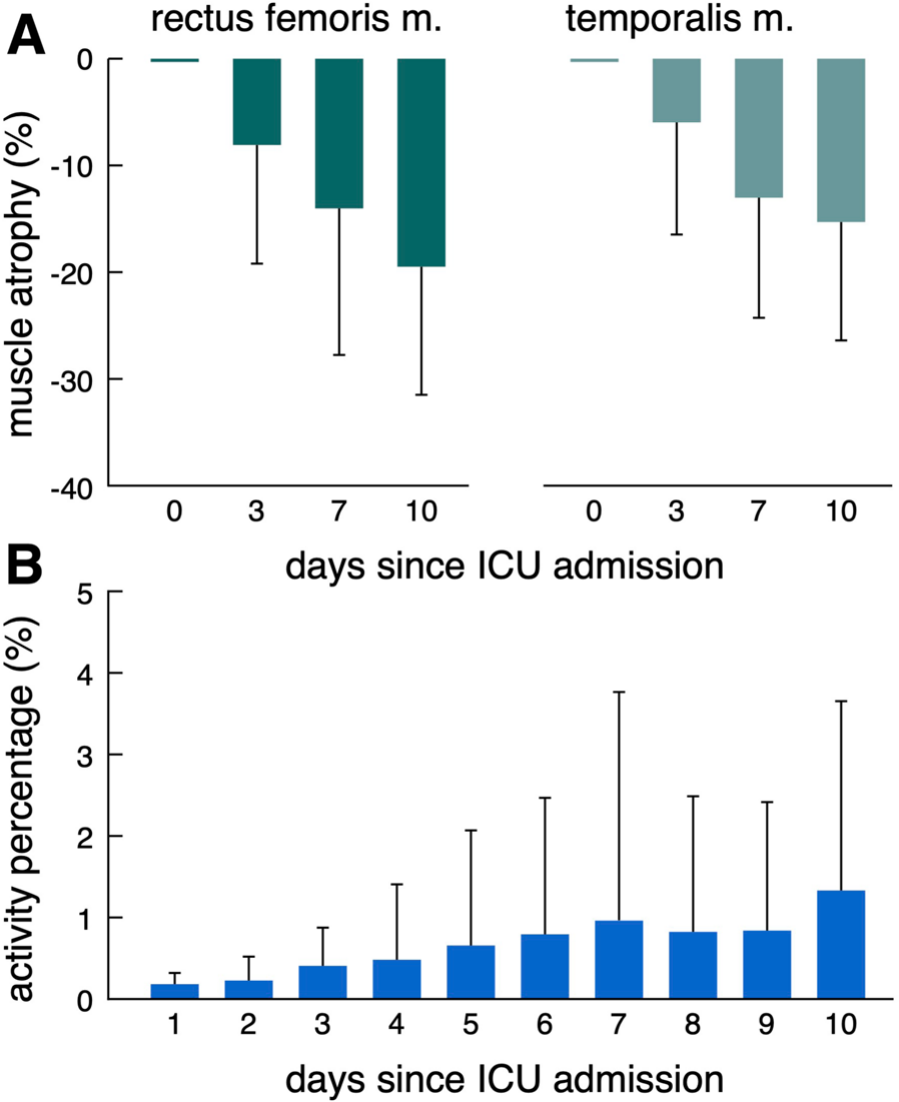
Active movement and muscle atrophy during ICU treatment. Muscle atrophy at days 3, 5 7 and 10 relative to day 0 for RFM and TM (**A**). Proportion of active movement (%active) over time (**B**). ICU = Intensive Care Unit;

Muscular atrophy as measured with ultrasound was more pronounced in RFM compared to TM (-19.5% (SD 12.0) versus -15.3% (SD 11.1) at day 10) (Fig. 4A). Active movement of NICU patients as indicated by proportion of active movement (%active) over time is infrequent, particular at early stages of the ICU stay. While mean %active stays low over the entire time, some patients exhibit higher activity starting around day 3. (Fig. 4B). Compared to healthy individuals, NICU patients demonstrate a significant reduction in active movement (%active: healthy individuals 13.3 (SD 0.8) vs. NICU patients 0.84 (SD 1.08), *p* < 0.001) (Supplementary Tables 2 and 4). No adverse events were observed in association with the placement of accelerometers in the ICU setting (Supplementary Table 3).

### Predictive models for muscle atrophy

**Fig. 5:**
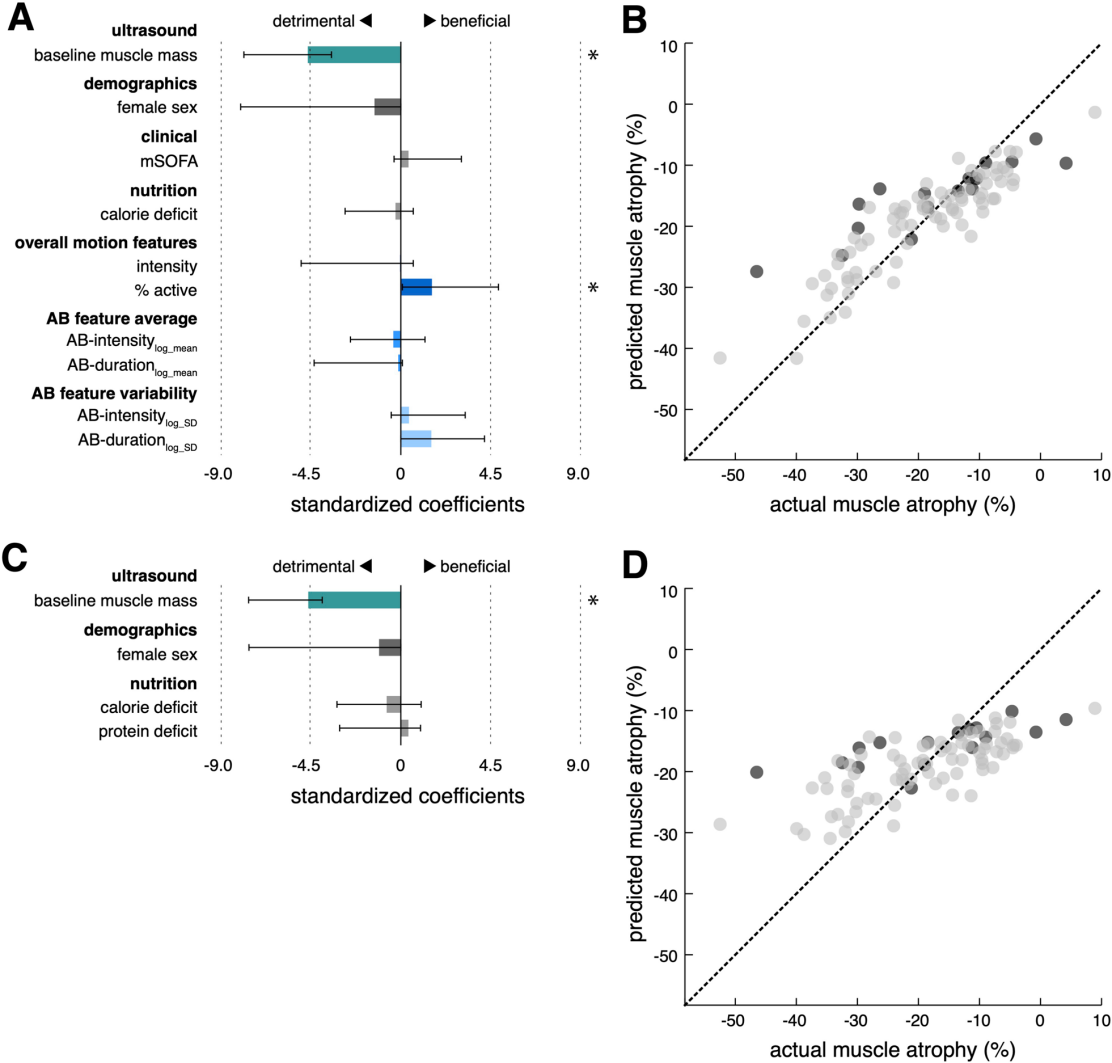
Prediction of MRF muscle atrophy with and without movement features. Standardized coefficients and 95% confidence intervals (asterisks indicate significant predictors) of the regularized regression models with (model_movement+_, **A**) and without movement features (model_movement−_, **C**). Out of all demographic (age, sex), clinical (baseline RFM muscle mass, mSOFA), nutritional (calorie deficit, protein deficit) and movement variables (intensity, %active, AB-intensity_log-mean_, AB-duration_log-mean_, AB-intensity_log-SD_, AB-duration_log-SD_), the depicted 10/12 independent variables for model model_movement+_ and 4/6 independent variables for model_movement−_ were selected for the final models, respectively. Significant predictors in model_movement+_ included baseline RFM muscle mass (beta − 5.1, 95% confidence interval (95% CI) − 7.9 to − 3.8) and %active (beta 1.6, 95% CI 0.1 to 4.9). For model_movement-_, only baseline RFM muscle was found as a statistically significant predictor (beta − 4.6, 95% CI − 7.6 to − 3.9). Scatter plots with regression line of predicted versus actual muscle wasting (grey dots: training data; black dots: unseen test data) for model_movement+_ (**B**) and model_movement-_ (**D**), respectively (R^2^: 0.79 vs. 0.55, RMSE: vs. 8.4 vs. 10.7 mm; MAE: 6.2 vs. 8.0 mm). mSOFA = SOFA without GCS;

The machine learning model based on a total of 12 demographic, clinical, nutritional and accelerometer-derived variables selected baseline RFM muscle mass (beta − 5.1, 95% confidence interval (95% CI) − 7.9 to − 3.8) and %active (beta 1.6, 95% CI 0.1 to 4.9) to explain 79% (R^2^ = 79%) of the occurring variance in muscle wasting in an unseen test data set (Fig. 5, A and B). Thus, for every standard deviation increase in baseline RFM (2.6 mm), RFM thickness at day 10 is estimated to decrease by another 5.1 percentage points (49.5% relative change). In contrast, a standard unit increase in %active (1.1%) is projected to result in 1.6 percentage points less RFM atrophy at day 10 (relative change 15.5%). Ignoring movement features as predictors for RFM muscle atrophy results in substantially worse model performance (R^2^ = 55%) (Fig. 5C, Supplementary Table 6). RFM atrophy was not significantly different between immobile limbs with upper motor neuron lesions (UMNL) and immobile limbs without UMNL. However, muscle atrophy was markedly decreased in limbs with active movement (Supplementary Fig. 2). Thigh-fixed accelerometer data did not contribute significantly to a model predicting TM atrophy (Supplementary Table 5).

## Discussion

In this prospective cohort study, we used thigh-fixed accelerometers to establish movement features as predictive biomarkers for muscle atrophy in neurocritical care patients. Proportion of active movement (%active) demonstrated a significant protective effect against muscle wasting and improved the precision of muscle atrophy prediction in an unseen test data set. To the best of our knowledge, this is the first quantifiable and validated measure that provides information on the relative importance of inactivity for muscle atrophy in critically ill patients.

It is crucial to distinguish between immobility and inactivity, especially within the ICU context, as inactivity can occur despite mobilization efforts due to a lack of active patient participation (passive movement). To address this, we excluded periods such as physiotherapy, intrahospital transports, and patient positioning, from our analysis. Therefore, we consider the movement features as surrogates for activity rather than measures of mobility. Importantly, and as a limitation of this approach, movement sensors are unable to capture any muscle activity without movement via isometric contractions (active immobility).

The relevance of inactivity, as compared to immobility, as the variable of interest in this context is further exemplified by clinical trials on electrical muscle stimulation (EMS) and interventions focusing on early (passive) mobilization. The current evidence highlights the efficacy of EMS [23, 24], whereas mobilization trials demonstrated limited efficacy and raised safety concerns [25–31]. While the latter often involve passive mobilization without genuine patient activity, EMS generates muscle activity without requiring mobility. Considering the data, a reasonable strategy to prevent muscle atrophy in critically ill patients may involve first measuring the extent of active movement with accelerometers to identify those at risk, and subsequently promoting activity (with or without mobilization) based on the patient's stability.

The pathophysiology of mechanical unloading leading to atrophy has so far only been systematically studied and quantified outside the ICU. Studies with cast immobilization of the lower extremity for two weeks in healthy adults and examination of astronauts after 8 days of space flight revealed a 5% and 6% decrease in quadriceps muscle mass, respectively [32, 33]. A recent meta-analysis analyzing the general ICU population estimated muscle atrophy to be around 16% at day 10 for RFM [34]. In comparison, our data demonstrate a more pronounced rate of RFM atrophy, showing a 19.5% decrease by day 10. While additional factors such as CIP, CIM, and CIPNM certainly contribute to the higher rate of atrophy in ICU patients, the residual activity in cast immobilization (via isometric contractions) and during space flight (via active movement with reduced muscle activity) may also account for the observed differences.

Given that no or passive movement was described in more than 70% of patients in the first 48 h, and still more than 40% after two weeks, in the TEAM trial [26], it is plausible to assume that inactivity also significantly contributes to ICUAW in the general ICU population. Yet, as disorders of consciousness and focal-neurological deficits are major barriers to mobilization and activity [26], this might be even more relevant for neurointensive care patients. Although our accelerometer data are not directly comparable to the ICU mobility scale used in the TEAM trial, it indicates extremely infrequent periods of active movement for most patients over a 10-day observation period, reaching only 6% (0.84/13.3) of the activity level of healthy individuals. These numbers are parallelled by data from González-Seguel et al., who found mechanically ventilated patients to be inactive during the ICU stay in over 96% of the time [35]. This, coupled with the prominence of movement features as predictors of muscle atrophy in our prospective cohort, further strengthens the significance of inactivity in (neuro-) critical care. Other studies within the ICU have investigated accelerometry primarily in the context of sleep, circadian rhythm, and sedation levels. However, these studies exhibit limitations, such as narrow observation periods and the absence of well-defined thresholds for activity measurement [36–39].

Accelerometer-derived data have also been validated as biomarkers for muscle atrophy outside the ICU setting. In a study with almost 500 elderly participants, Sanchez-Sanchez et al. investigated the association of physical activity as measured with hip-worn accelerometers and sarcopenia. Here, higher physical activity correlated with better performance in sarcopenia-related scores [40]. Similarly, Foong et al. showed a positive association of accelerometer-derived physical activity with muscle mass and muscle strength [41].

The exercise stimulus, as the ultimate determinant for activity, can be delineated into two primary variables: volume and intensity. In exercise physiology, the volume of exercise is traditionally quantified by the number of repetitions performed, while intensity is commonly measured by the force exerted during exercise [42]. In our ICU cohort, we utilized proportion of active movement (%active) and AB-duration_log-mean_ as proxies for exercise volume. For critically ill patients, the force generated cannot be measured pragmatically. We therefore introduced movement intensity (resultant acceleration magnitude) as a surrogate of exercise intensity. The LASSO regularization used to address the high number and potential co-linearity of parameters revealed %active as an approximation of exercise volume as a relevant predictor, while surrogates of intensity were not selected. Thus, intensity may either be irrelevant considering the uniform force generated by patients moving against gravity and not against resistance, or movement intensity is not a valid biomarker of the exercise intensity.

Besides proportion of active movement, baseline muscle mass was predictive of muscle atrophy. This finding is in line with studies in healthy participants. Here, higher age with lower baseline muscle mass showed significantly less pronounced atrophy. However, older participants with lower baseline muscle mass suffered from greater loss of muscle strength after immobilization [43–45]. Furthermore, variations in atrophy can be observed across muscle and fiber types. Anti-gravity muscles with high proportions of slow type 1 fibers, such as RFM, seem to exhibit a selective vulnerability [45], which is in line with our data demonstrating pronounced atrophy of RFM over TM.

The strengths of our study include the selection of a neurointensive care population devoid of frailty, specifically targeting individuals at high risk of muscle atrophy without pre-existing muscle wasting. However, the study's focus on neurocritical care may limit its generalizability and further research is needed to confirm the applicability of our results to more diverse patient populations. The extent of neurogenic atrophy mediated via various damage to the lower motor neuron was not explicitly measured in our study but can be assumed to be minimal given the demographics of our cohort. As that general ICU cohorts also experience lower motor neuron lesions due to critical illness or its treatments, this confounder is likely to be similar across groups. For UMNL, we do not expect neurogenic atrophy, as it does not result in direct muscle denervation. Supporting this pathophysiological hypothesis, we could not observe any difference in the extent of muscle atrophy between immobile limbs with upper motor neuron lesions (UMNL) and immobile limbs without UMNL. However, muscle atrophy was markedly decreased in limbs with active movement, suggesting no role for UMNL as a mediator for atrophy. We ensured high data quality by filtering out passive mobilization and prospectively collecting clinical data. Furthermore, our analysis is underpinned by a strong statistical framework leveraging machine learning to identify the most important predictors and using unseen data to validate these findings. Further limitations of our study are primarily rooted in the fact that muscle morphology does not necessarily equate to function. We used muscle ultrasound, a widely adopted and validated surrogate for ICUAW [34, 46, 47], instead of the Medical Research Council Sum Score (MRC-SS), as the latter is often deemed infeasible in the general ICU cohort, and even more so in neurocritical care. Additionally, we decided against including measures of the upper extremities because muscle volume is challenging to determine via ultrasound due to variability in muscle thickness relative to positioning, difficult anatomical landmarks, and less pronounced atrophy compared to the lower extremities. Instead, we focused on the RFM as the established sonographic gold standard, along with the TM as a muscle group unrelated to the movement captured by the accelerometers.

## Conclusion

Active movement, as a surrogate of muscle activity, can be quantified using non-invasive, thigh-fixed accelerometers and adds value for the prediction of muscle atrophy in neurocritical care patients. Establishing movement-derived biomarkers enables better phenotyping of ICUAW, thereby providing a foundation for tailored interventions and should be included as covariates in trials on ICUAW in the future. Studies addressing the external validity of these findings beyond the neurointensive care unit are warranted.

### Supplementary Information

Supplementary Figure 1. Accelerometer placement. Figure 2. Influence of movement and upper motor neuron lesion on muscle atrophy. Table 1. Reliability of RFM ultrasound measurements. Table 2. Negative and positive controls. Table 3. Adverse events of accelerometer placement. Table 4. Accelerometer data. Table 5. Linear regression model for TM atrophy. Table 6. Performance metrics of models with and without movement features.

## Data Availability

The datasets used and/or analyzed during the current study are available from the corresponding author on reasonable request. Matlab code for analyzing motion features is available at https://github.com/DSGZ-MotionLab/ICU_motion_analysis/.

## Abbreviations

AB: Activity bout
APACHE II: Acute physiology and chronic health evaluation II
BCa: Bias-corrected and accelerated
BMI: Body mass index
CI: Confidence interval
CIM: Critical illness myopathy
CIP: Critical illness polyneuropathy
CIPNM: Critical illness polyneuromyopathy
EE: Energy expenditure
EMS: Electrical muscle stimulation
ESPEN: European Society of Parenteral and Enteral Nutrition
GCS: Glasgow Coma Scale
GEE: Generalized Estimating Equations
GLMM: Generalized linear mixed model
GOSE: Glasgow Outcome Scale—Extended
HLF: High/low pass filter
ICU: Intensive care unit
ICUAW: Intensive care unit acquired weakness
LASSO: Least Absolute Shrinkage and Selection Operator
LOS: Length of stay
MINCE: Metabolism and nutrition in neurointensive care
MRC-SS: Medical Research Council Sum Score
mSOFA: Modified SOFA
NCS: Nerve conduction studies
NICU: Neurointensive care unit
NUTRIC: Nutrition risk in the critically ill
PICS: Post intensive care syndrome
pmRS: Premorbid modified Rankin scale
QQ: Quantile–Quantile
RFM: Rectus femoris muscle
SMA: Signal magnitude area
SOFA: Sequential organ failure assessment
TM: Temporalis muscle
UMNL: Upper motor neuron lesion
