# Supplementary material for: Accelerometer-derived movement features as predictive biomarkers for muscle atrophy in neurocritical care: a prospective cohort study
Source: Crit Care. 2024 Aug 31;28:288. doi: 10.1186/s13054-024-05067-y (PMC11366141; doi:10.1186/s13054-024-05067-y)
Supplement: Supplementary file 1 — Supplementary Figure 1. Accelerometer placement. Figure 2. Influence of movement and upper motor neuron lesion on muscle atrophy. Table 1. Reliability of RFM ultrasound measurements. Table 2. Negative and positive controls. Table 3. Adverse events of accelerometer placement. Table 4. Accelerometer data. Table 5. Linear regression model for TM atrophy. Table 6. Performance metrics of models with and without movement features. [file 13054_2024_5067_MOESM1_ESM.docx]

**Accelerometer-derived movement features as predictive biomarkers for muscle atrophy in neurocritical care –
a prospective cohort study**

Moritz L. Schmidbauer^1#^, Timon Putz^1#^, Leon Gehri^1^, Luka Ratkovic^1^, Andreas Maskos^1^, Julia Zibold^1^, Johanna Bauchmüller,^1^ Sophie Imhof,^1^ Thomas Weig^2^, Max Wuehr^1,3†^ and Konstantinos Dimitriadis ^1†^

^#†^ MLS, TP, MW and KD contributed equally

**Affiliations:**

1. Department of Neurology, LMU University Hospital, LMU Munich
2. Department of Anaesthesiology, LMU University Hospital, LMU Munich
3. German Center for Vertigo and Balance Disorders (DSGZ), LMU University Hospital, LMU Munich

**Correspondence:**Moritz Schmidbauer MD, Department of Neurology, University Hospital LMU Munich
Tel ++49-4400-72825, [moritz.schmidbauer@med.uni-muenchen.de](mailto:moritz.schmidbauer@med.uni-muenchen.de)

**Keywords:**

ICU, ICUAW, sarcopenia, muscle atrophy, accelerometer, machine learning

## SUPPLEMENTARY DATA

**Supplementary Figure 1**
Accelerometer placement


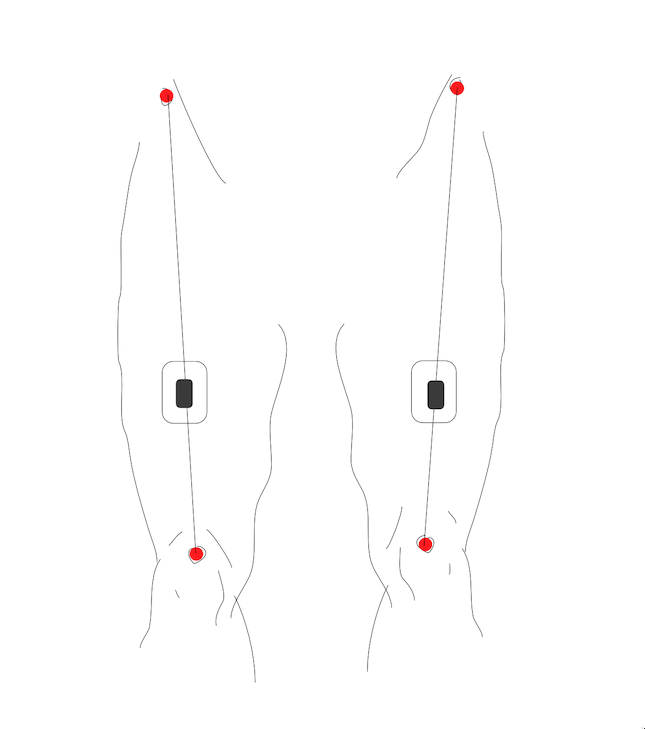


**Supplementary Figure 2**


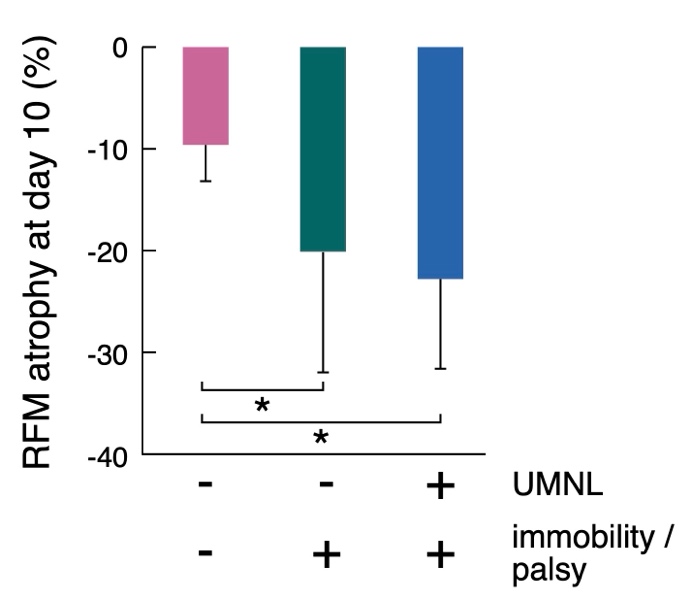
Influence of movement and upper motor neuron lesion on muscle atrophy

Immobility/palsy was considered positive if the respective limb exhibited a palsy (MRC <5) at day 10. UMNL was identified using clinical and neuroimaging data. Pairwise comparisons were performed using Generalized Estimating Equations (GEE) with post hoc testing and Bonferroni adjustments. UMNL-/inactivity- versus UMNL-/inactivity+, p = 0.016, UMNL-/inactivity- versus UMNL+/inactivity+: p < 0.001

UMNL-/inactivity+ versus UMNL+/inactivity+: p = 1.000.

UMNL – upper motor neuron lesion; MRC – Medical Research Counsil

**Supplementary Table 1**

Reliability of RFM ultrasound measurements

| Standard Deviation (SD), mm | Intraclass Correlation Coefficient (ICC) | Standard Error of Measurement (SEM), mm |
| --- | --- | --- |
| 0.26 | 0.99 | 0.02 |

ICC was calculated using the ICC(1,k) model to evaluate the consistency of measurements taken on the same subject across k repetitions (within-subject variability).

**Supplementary Table 2**
Negative and positive controls

|  | | Proportion of active movement* | | AB-duration (log mean) | | AB-intensity (log mean) | |
| --- | --- | --- | --- | --- | --- | --- | --- |
| Healthy individuals (n=3), mean (SD) | 13.3 (0.8) | | 2.47 (0.05) | | -1.27 (0.05) | |  |
| Static placement (n=1), mean (SD) | | 0 (-) | | 0 (-) | | 0 (-) | |

SD – Standard Deviation; AB – activity bout;

**Supplementary Table 3**
Adverse events of accelerometer placement

| **Adverse events** | **n = 53** |
| --- | --- |
| Allergic reaction, n (%) | 0/0 (0%) |
| Pressure ulceration, n (%) | 0/0 (0%) |
| Discomfort (pain/itching), n (%) | 0/0 (0%) |

**Supplementary Table 4**
Accelerometer data

| Parameter | n = 91 |
| --- | --- |
| Total hours of recording | 15,643.1 |
| Total hours cut from sensor data | 299.0 |
| Hours of sensor recording per patient, mean (SD) | 302.6 (48.4) |
| Hours cut from recording per patient, mean (SD) | 5.8 (5.2) |
| Overall intensity (SMA, g), mean (SD) | 0.011 (0.005) |
| Proportion of active movement (SMA > 0.135g, %) relative to total recording period (%active), mean (SD) | 0.842 (1.083) |
| AB-duration (log mean), mean (SD) | 1.900 (0.087) |
| AB-duration variability (log SD), mean (SD) | 0.461 (0.082) |
| AB-intensity (log mean), mean (SD) | -1.626 (0.064) |
| AB-intensity variability (log SD), mean (SD) | 0.286 (0.047) |

SMA = signal magnitude area; AB = activity bout; SD = standard deviation;

**Supplementary Table 5**Linear regression model for TM atrophy

| Variable | Estimate | SD | p-value |
| --- | --- | --- | --- |
| Intercept | -13.99 | 21.70 | 0.52 |
| Age | 0.14 | 1.73 | 0.10 |
| Female sex | 4.36 | 1.22 | 0.23 |
| Modified SOFA (mSOFA, without GCS) | 0.29 | -0.69 | 0.50 |
| Baseline TM thickness | -0.92 | 1.48 | 0.54 |
| Calorie deficit | 2.34 | 11.13 | 0.83 |
| Protein deficit | -7.193 | 11.81 | 0.55 |
| Proportion of active movement | 0.09 | 2.00 | 0.96 |

GCS = Glasgow Come Scale; TM = temporal muscle; SD = standard deviation;

**Supplementary Table 6**
Performance metrics of models with and without movement features

| Metric | Model 1 (including movement features) | Model 2 (excluding movement features) |
| --- | --- | --- |
| Mean Squared Error (mm^2^) | 70.0 | 113.8 |
| Root Mean Squared Error (mm) | 8.4 | 10.7 |
| Mean Absolute Error (mm) | 6.2 | 8.0 |
| R-Squared (%) | 79 | 55 |
